# The Quantitative Criteria Based on the Fractal Dimensions, Entropy, and Lacunarity for the Spatial Distribution of Cancer Cell Nuclei Enable Identification of Low or High Aggressive Prostate Carcinomas

**DOI:** 10.3389/fphys.2016.00034

**Published:** 2016-02-11

**Authors:** Przemyslaw Waliszewski

**Affiliations:** ^1^Department of Urology, Alb Fils KlinikenGoeppingen, Germany; ^2^The Bȩdlewo Institute for Complexity ResearchPoznań, Poland

**Keywords:** fractals, complexity, Renyi dimensions, entropy, lacunarity, tumor aggressiveness, grading, prostate carcinoma

## Abstract

**Background:** Tumor grading, PSA concentration, and stage determine a risk of prostate cancer patients with accuracy of about 70%. An approach based on the fractal geometrical model was proposed to eliminate subjectivity from the evaluation of tumor aggressiveness and to improve the prediction. This study was undertaken to validate classes of equivalence for the spatial distribution of cancer cell nuclei in a larger, independent set of prostate carcinomas.

**Methods:** The global fractal capacity *D*_0_, information *D*_1_ and correlation *D*_2_ dimension, the local fractal dimension (LFD) and the local connected fractal dimension (LCFD), Shannon entropy *H* and lacunarity λ were measured using computer algorithms in digitalized images of both the reference set (*n* = 60) and the test set (*n* = 208) of prostate carcinomas.

**Results:** Prostate carcinomas were re-stratified into seven classes of equivalence. The cut-off *D*_0_-values 1.5450, 1.5820, 1.6270, 1.6490, 1.6980, 1.7640 defined the classes from C1 to C7, respectively. The other measures but the *D*_1_ failed to define the same classes of equivalence. The pairs (*D*_0_, LFD), (*D*_0_, *H*), (*D*_0_, λ), (*D*_1_, LFD), (*D*_1_, *H*), (*D*_1_, λ) characterized the spatial distribution of cancer cell nuclei in each class. The co-application of those measures enabled the subordination of prostate carcinomas to one out of three clusters associated with different tumor aggressiveness. For *D*_0_ < 1.5820, LFD < 1.3, LCFD > 1.5, *H* < 0.7, and λ > 0.8, the class C1 or C2 contains low complexity low aggressive carcinomas exclusively. For *D*_0_ > 1.6980, LFD > 1.7644, LCFD > 1.7051, *H* > 0.9, and λ < 0.7, the class C6 or C7 contains high complexity high aggressive carcinomas.

**Conclusions:** The cut-off *D*_0_-values defining the classes of equivalence were validated in this study. The cluster analysis suggested that the number of the subjective Gleason grades and the number of the objective classes of equivalence could be decreased from seven to three without a loss of clinically relevant information. Two novel quantitative criteria based on the complexity and the diversity measures enabled the identification of low or high aggressive prostate carcinomas and should be verified in the future multicenter, randomized studies.

## Synopsis

The spatial distribution of cancer cell nuclei in digitalized histological images of prostate carcinomas reflects complex intercellular interactions within malignant tumor underlying its biological aggressiveness. Since this distribution can be characterized by a number of complexity and diversity measures, those measures define the quantitative criteria for low complexity carcinomas with low aggressiveness or for high complexity carcinomas with high aggressiveness.

## Introduction

Histological evaluation of tumor aggressiveness, that is a potential of cancer cells for local growth and metastasis formation, is a key element in both diagnosis and prognosis of any malignancy. In the case of prostate carcinomas, pathologists developed about 40 subjective grading systems scaling aggressiveness of those tumors (reviewed in Humphrey, 2003). The Gleason system (Gleason, 1977) is the most commonly used one (Humphrey, 2004). It was validated with a database of a few thousand prostate cancer patients. Since its revision in 2005 and in 2010, the Gleason system comprises three patterns of tumor growth called the Gleason grades (Epstein et al., 2005; Epstein, 2010). The Gleason score is calculated as a sum of two or, sometimes, three Gleason grades representing most predominant patterns of growth in a given carcinoma. This information co-determines the risk stratification of cancer patients. According to that stratification, cancer patients will undergo follow-up or will be treated. That latter option comprises a choice of the optimal therapy, extent of surgical resection with or without nerve sparing or dosing of radiation therapy (Epstein et al., 2005; Epstein, 2010; Lotan and Epstein, 2010).

A prostate biopsy is a procedure to obtain small samples of prostate tissue for the microscopic examination using a thin needle. Owing to both the limitations in obtaining a representative tissue specimen and a low specificity of this procedure, there is no good correlation between the grading score of the prostate carcinomas in biopsy tissues and in prostatectomy specimen (Fine et al., 2012). Using a prostate biopsy, one cannot predict the existence of insignificant (indolent) cancer, that is cancer without a significant influence on survival (Epstein et al., 1994), the final histological grade, size of tumor, its extracapsular extension, the existence of positive margins or lymph node involvement (D'Amico et al., 2000; Boccon-Gibod et al., 2005; Fine et al., 2012). In addition, the modification of the criteria in the Gleason system (Epstein et al., 2005; Epstein, 2010) caused both a discrepancy in tumor grading and difficulties in comparison of treatment results for patients treated before and after the change (Lotan and Epstein, 2010). Although tumor aggressiveness is a key parameter in the prediction models, the subjectivity of histological evaluation is a source of the prediction weakness. Human eye is not able to evaluate correctly many details in tumor images, such as the ratio of cells bound in glands to the number of infiltrating cancer cells, or some configurations, such as the co-existence of glands of different size and shape. It is a challenge for pathologists to match the images to the definition of a grade, especially in the borderline cases (reviewed in Montironi et al., 2013). The subjective tumor grading has a significant inter- and intraobserver variability in the range of 38–80% (Nguyen et al., 2004; van der Kwast et al., 2010; McKenney et al., 2011; Netto et al., 2011; Egevad et al., 2013; Berney et al., 2014) and the coefficient κ for interobserver agreement 0.15–0.7 (McLean et al., 1997; Allsbrook et al., 2001; van der Kwast et al., 2010; McKenney et al., 2011; Scott Lucia et al., 2013). Even though some DNA-, RNA-, or protein biomarkers were co-applied, it did not improve the accuracy of grading. The value of the correlation coefficient was low in the range of 0.1–0.6 (McDunn et al., 2013; Pin et al., 2013). A novel diagnostic tool, prostate magnetic resonance imaging reinforced by the multiwavelet filters was supposed to enable the automated diagnosis of prostate carcinoma. The results remain nonspecific. Biopsies must be performed to evaluate tumor aggressiveness nonetheless (reviewed in Turkbey and Choyke, 2012).

A decision whether a prostate cancer patient should be treated or not depends on the fulfillment of two criteria [Klein, 2014; Mottet et al., 2015; National Comprehensive Cancer Network (NCCN), 2015]. The first one is the patient's overall life expectancy determined by age, health status, and comorbidities. In particular, patients older than 75 years, with life expectancy less than 10 years, in a reduced health status or with serious comorbidities will not be treated with the intention to cure. This category of patients will be offered a program of watchful waiting with a follow-up schedule. A palliative treatment can be initiated if local or metastatic progression with clinical symptoms occurs. The second criterion plays a pivotal role in the risk assessment of cancer progression. It considers three parameters (Partin et al., 1997), such as biological aggressiveness of tumor described by the subjective tumor grading according to Gleason (Gleason, 1977; Humphrey, 2003, 2004; Epstein et al., 2005; Epstein, 2010), PSA concentration in serum (Stamey et al., 1989; D'Amico et al., 2004, 2005; Thompson et al., 2006; ElShafei et al., 2013; Wu et al., 2014), and tumor stage (Prout et al., 1980; Petros and Catalona, 1992). The statistical accuracy of the nomograms based on those parameters approximates 70% only (Partin et al., 1997; Miller et al., 2006; Thompson et al., 2006; Klotz et al., 2010).

According to the clinical guidelines [Klein, 2014; Mottet et al., 2015; National Comprehensive Cancer Network (NCCN), 2015], low risk cancer patients with PSA < 10 ng/ml, Gleason score of 6 or less, and pT1-2a stage do not need any treatment, nor an adjuvant therapy. Despite of those criteria, the overtreatment occurs at the indolent stadium of the disease in about 55% of patients (Miller et al., 2006; Danzig and McKiernan, 2014; Daskivich et al., 2014). Urologists recommend frequently treatment even for 80-year-old patients with limited life expectancy (Dall'Era et al., 2008; Albertsen, 2010; Danzig and McKiernan, 2014; Daskivich et al., 2014). Rather, those patients should be followed-up to identify local tumor progression and aggressive metastatic disease in the long term (Klotz, 2006; Denis, 2007; Dall'Era et al., 2008; Albertsen, 2010); phenomena especially frequent about 15 years from the diagnosis (Albertsen et al., 2005; Cooperberg et al., 2011; Cooperberg, 2015; Klotz et al., 2014). The same strategy might also be offered to some patients with intermediate risk of progression (PSA 10–20 ng/ml, Gleason score 7, pT2b-2c) (Cooperberg et al., 2011). Unfortunately, only up to 49% of eligible patients participate in the follow-up programs (Womble et al., 2015). The lack of the histological, molecular or clinical criteria for the unequivocal identification of the low risk cancer patients increases a fear for tumor progression among those involved in active surveillance as well as among those who already underwent some kind of aggressive therapy, without even knowing if they needed it (Womble et al., 2015).

Patients with high-risk (PSA >20 ng/ml, Gleason score 8–10, stage T3a) or very high risk carcinomas (stage T3b–T4 N0 or any T N1) should be treated aggressively with all available modalities, such as surgery, radiotherapy, brachytherapy, or hormonal therapy, even though a choice of a modality seems to increase survival in about 10% of patients (Heidenreich et al., 2014). For example, SEER, a population-based cohort study of 66,717 patients 66 years of age or older with localized prostate cancer demonstrated that primary hormonal therapy did not improve long-term overall or disease-specific survival (Lu-Yao et al., 2014).

The relevant question, whether a given prostate carcinoma is an indolent, low grade, low risk malignancy rather than a high grade, high risk one can be answered only if prostate gland is removed surgically. Although this answer appears after the initiation of the aggressive treatment, it is still important to get the unequivocal evaluation of tumor aggressiveness based on some objective, quantitative criteria. Those criteria might help to improve the accuracy of prognosis, to plan more individualized therapy, or to facilitate a search for some features of cancer cells that would improve predictions made on the basis of biopsies. A variety of approaches, such as graph models (Altunbay et al., 2010), gland segmentation with morphometric analysis and application of probabilistic pairwise Markov models (Naik et al., 2008; Loeffler et al., 2012; Nguyen et al., 2012), color channel histograms, texture analysis, fractal algorithm with fractal code or multifeature analysis with a number of classifiers, such as Bayesian one (Doyle et al., 2007), multiwavelet method (Jafari-Khouzani and Soltanian-Zadeh, 2003; Candes et al., 2006; Huang and Lee, 2009; Hong-Jun et al., 2011; Veltri et al., 2012; Lopez and Again, 2013), k-NN one (Tabesh et al., 2005), support vector machine (Tabesh et al., 2007), or linear discriminant analysis method (Tabesh et al., 2005, 2007) were proposed for the automated diagnosis of prostate carcinoma or for the evaluation of tumor aggressiveness. Those statistically elegant and mathematically advanced approaches resulted in the maximal accuracy of 78–81% for grading in the case of multiwavelet method (Tabesh et al., 2005, 2007). The accuracy of 95–97% was reported only for the low number of carcinomas (Lopez and Again, 2013). This discrepancy between the Gleason grading and the quantitative classifications generated by the sophisticated computer algorithms had at least one reason. Two different categories of data, the subjective and the objective one, should not be compared each other if the subjective tumor grading according to Gleason is chosen as the absolute frame of reference.

Since, there is no golden standard in pathology of adenocarcinomas that might be used as the objective frame of reference for the quantitative studies, a novel approach based on the fractal geometric model of prostate carcinomas and evaluation of complexity in the spatial distribution of cancer cell nuclei was proposed (Waliszewski, 2013; Waliszewski et al., 2014, 2015). Cell nuclei are fragments of the surface in two-dimensional histological images. They compose irregular, yet self-affine configurations (Waliszewski, 2013). The fractal geometrical model of prostate carcinomas introduced the idea of the distinct classes of equivalence called complexity classes (Waliszewski et al., 2015). Those classes were defined by the values of the global capacity fractal dimension *D*_0_. Using that model, all basic patterns of growth seen in prostate cancer were subordinated to the appropriate class. In particular, low grade prostate carcinomas with well-preserved glandular structure were re-stratified to the class C1, C2, or C3. High grade carcinomas with the predominating infiltrating patterns of growth were stratified to the class C6 or C7.

This study was undertaken to validate the classes of equivalence (Waliszewski et al., 2015) using utterly novel set of carcinomas than that used in the study (Waliszewski, 2013; Waliszewski et al., 2014, 2015). A question arises whether the additional complexity or diversity measures, such as the local fractal dimensions, Shannon entropy or lacunarity allow a similar stratification of prostate carcinomas into the same classes of equivalence? Is it possible to define some quantitative criteria based on all those parameters that define prostate carcinomas with low or high complexity of the spatial distribution of cancer cell nuclei associated with low or high tumor aggressiveness?

## Methods

### The renyi family of the global fractal dimensions

The Renyi family of the global spatial fractal dimensions comprises the capacity (*D*_0_), information (*D*_1_), and correlation (*D*_2_) dimension. The appropriate introduction to the theory of dimension, fractals, or the formal mathematical definitions of the fractal dimensions can be found elsewhere (Engelking, 1978; Czyz, 1994). Briefly, the capacity dimension *D*_0_ is defined as a relationship between the logarithm of a number of boxes *N*(ε) covering the geometric object and the logarithm of a box size ε (Equation 1; Vicsek, 1992; Kinsner, 2007):

(1) $$D_{0}=\underset{\varepsilon \to 0}{\lim}\frac{\log N(\varepsilon )}{\log\frac{1}{\varepsilon }}$$

The information dimension *D*_1_ measures how the average information needed to identify an occupied box, scales, as the scale of boxes gets smaller. The algorithm for the information dimension will search for the linear relationship between the logarithm of a box size (ε) and the logarithm of the probability *p* that a given box contains the element of the object (Equation 2; Baker and Gollub, 1996).

(2) $$D_{1}=\underset{\varepsilon \to 0}{\lim}\frac{-\langle \log p_{\varepsilon }\rangle }{\log\frac{1}{\varepsilon }}$$

The correlation dimension *D*_2_ measures the number of points *M* used to generate a representation of the fractal and the number of pairs of points closer than ε to each other. The correlation dimension is a probability measure that two pixels within the object are close to each other less than ε. The appropriate algorithm calculates not only if a given box is occupied by the pixel of the analyzed geometric object, but also how many pixels in the box are, and how close to each other they are (Equation 3):

(3) D2=limε→0,M→∞log(gεM2)log ε

in which *M* is a number of pixels of the analyzed geometric object and *g*_ε_ denotes a number of pairs of pixels that are closer to each other than ε (Grassberger and Proccacia, 1983; Baker and Gollub, 1996).

### Local connected fractal dimension

The local fractal dimension (LFD) is the fractal capacity dimension calculated according to Equation 1 for every pixel in the image. This dimension is a local and indirect measure of intercellular connectivity, i.e., interconnectedness which denotes the existence of complex, dynamic relationships in a population of cells leading to the spatial and temporal emergence of global features in the system that would never appear in a single cell existing out of the system (Waliszewski, 1997, 2005, 2009; Waliszewski et al., 1998, 2001; Waliszewski and Konarski, 2001).

The local connected fractal dimension (LCFD) characterizes local irregularities of geometry of heterogeneous geometrical objects, such as clusters of cancer cells present either in glands or in infiltrates. Instead of a single value of the global fractal capacity dimension *D*_0_ calculated for the entire image, one gets a set of values calculated for each pixel that belongs to the analyzed object. This is done according to the following Image J algorithm:

Step 1: choose a pixel P that belongs to the analyzed object and possesses eight neighbor pixels.
Step 2: define the local connected set of pixels by finding all the pixels connected to the pixel P within the increasing s-pixel-side window centered at P.
Step 3: count how many pixels N(s) of the analyzed object are within the window.
Step 4: use the least square method to compute the slope of the log-log curve composed by the coordinates [log(N(s), log(s); Landini et al., 1995].

### Entropy and the global information fractal dimension *D*_1_

The Shannon entropy *H* is a statistical measure of both the information content and diversity in a given cell configuration in the image. It can be used to characterize gray images or binary images of the spatial distribution of cancer cell nuclei. The Shannon entropy describes the following Equation (4):

(4) $$H=-\sum_{i}p_{i}\;\log_{2}\;p_{i}$$

In the above equation, *p*_*i*_ denotes the probability that the difference between two adjacent pixels of the image is equal to *i*, and is calculated from the histogram counts. Low entropy images, such as those containing mostly black background, e.g., in benign prostatic hyperplasia or in well-differentiated adenocarcinomas, have large numbers of pixels with the same zero value. The black image that contains no white pixels will have entropy equal zero.

The entropy *H*_*D*_ of a set with *D* dimensions is defined by the following Equation (5):

(5) $$H_{D}=-\sum_{i}p_{i}\;\log_{2}\;p_{i}+Dlog\;\varepsilon =H+Dlog\varepsilon$$

ín which *H* stands for the Shannon entropy (see Equation 4), ε denotes size. In consequence, entropy *H* is a linear function of log ε with slope *D* and intersection *H*_*D*_. It is important to notice that the slope *D* in the limit for ε = 0 becomes the global information fractal dimension *D*_1_ (see Equation 2; Sethna, 2011).

### Lacunarity

Cancer cells form various morphological patterns. The areas of the image complementary to the lumen of glands correspond to the gaps on the binary image. Investigating those gaps provides important information about homogeneity of cell distribution in malignant tumor. Lacunarity λ, that is, a degree of gappiness, inhomogeneity, or translational and rotational invariance in the image characterizes quantitatively those gaps as well as differences in their spatial distribution between the patterns of growth. This parameter can also quantify differences between self-similar structures having the same global fractal dimension (Plotnick et al., 1996).

In general, lacunarity λ is equivalent to the square of the coefficient of variation, CV, and expresses a relationship between standard deviation σ and mean μ for the pixels in a given area of an image, and a number of pairs of pixels *g* that are closer to each other than ε:

(6) $$\lambda_{\epsilon ,g}={\left(CV_{\epsilon ,g}\right)}^{2}={\left(\frac{\sigma_{\epsilon ,g}}{\mu_{\epsilon ,g}}\right)}^{2}$$

More specifically, lacunarity λ is a relationship between variation for the function *P*(*m, r*), that defines probability of the localization of the pixels *m* in the square of size *r* to the square of the mean value of that function (Equation 7):

(7) $$\lambda (r)=\frac{\sum_{m=1}^{r^{2}}m^{2}P(m,r)-{\left(\sum_{m=1}^{r^{2}}mP\left(m,r\right)\right)}^{2}}{{\left(\sum_{m=1}^{r^{2}}mP\left(m,r\right)\right)}^{2}}$$

The computer algorithms that calculate the mean value of lacunarity for images use the same principle as in the case of the global fractal dimensions, i.e., they analyze the digital image from different scaled levels of resolution to examine how certain geometric features change with the size of the element used to inspect the image. The first approach, called box counting, is identical with the principle underlying the capacity dimension (see above) in the sense that the box for each ε is placed as though it were part of a grid overlaid on the image so that the box does not overlap itself. In the second one, called the sliding box algorithm, the box is slid over the image so that it overlaps itself and the sliding box lacunarity is calculated. The values are usually expressed as logarithms. Lacunarity analyses using the types of values discussed above have shown that data sets extracted from geometric fractals, or from patterns that change little when rotated have low lacunarity. The larger and more irregular are the gaps in the image, as those seen in glands in benign prostatic hyperplasia, the greater is the value of lacunarity. In some instances, fractal dimensions and values of lacunarity can be correlated, but it does not hold for all types of patterns and measures of lacunarity.

### Patients, preparation of images, and isolation of cell nuclei

This study was performed according to the ethical standards outlined in the WMA Declaration of Helsinki “Ethical Principles for Medical Research Involving Human Subjects” (http://www.wma.net). The data bank was constructed and analyzed according to the ethical requirements of the Ethics Committee for the Medical Faculty of the Justus-Liebig University in Giessen, Germany (ethical vote 49/05). Since this project was based solely on computer-aided image analysis of the digitalized anonymous tissue slides of prostate carcinomas, no written consent from the prostate cancer patients was necessary. The surgical procedures were performed from 2007 to 2010. Tissues were stained with hematoxylin and eosin as well as with the appropriate antibodies against the markers, i.e., PSA (DAKO, Germany) and AMACR (DAKO, Germany) for prostate cancer cells, cytokeratin 34βE12 (Leica Biosystems, Canada) for basal cells in order to confirm the diagnosis (Kristiansen and Epstein, 2014), and classified by the Gleason criteria (Gleason, 1977; Humphrey, 2003, 2004; Epstein et al., 2005; Epstein, 2010).

A reference set of prostate carcinomas comprised cases representing the homogeneous patterns with the Gleason score 3+3 = 6 defined as a regular gland architecture with no basal cells and no areas of cellular infiltration (*n* = 20), the Gleason score 4+4 = 8 defined as dominating small glands spread freely in the prostate tissue or organized in the cords (*n* = 20), and the Gleason score 5+5 = 10 defined as a random infiltration of the prostate tissue by cancer cells without any glands (*n* = 20), and benign prostatic hyperplasia (*n* = 10). We analyzed and re-stratified a test set of prostate carcinomas comprising 208 carcinomas with the following Gleason score: 3+3 = 6 (*n* = 70), 3+4 = 7*a* (*n* = 18), 4+3 = 7*b* (*n* = 14), 4+4 = 8 (*n* = 23), 4+5 = 9 (*n* = 28), 5+4 = 9 (*n* = 20), 5+5 (*n* = 35). All prostate carcinomas were subordinated to the appropriate subsets called structural Gleason classes using the Gleason criteria. We also analyzed benign prostatic hyperplasia (*n* = 20). None of the patients had lymphatic or distant metastases.

Histological slides of prostate carcinomas stained with hematoxylin and eosin were digitalized using both a microscope Axioskop 5.0 with the halogen lamp 12V 50W 2800K 950 lm and the objective Plan Neofluar 20x with the numerical aperture 0.5, Zeiss, Germany and a camera Nikon Coolscope, Japan. The optimal conditions for the magnification (20x) and lighting intensity (41.19 × 10^6^ lx) were chosen in such a manner that the values of the capacity fractal dimension for the test image were in the plateau-area of the test curve (data not shown).

The color images in the bmp format with 1240 × 1000 pixels and resolution 150 × 150 dpi were first resized to the jpg format with 648 × 432 pixels and resolution 150 × 150 dpi. Cancer cell nuclei were isolated electronically using a package Definiens Tissue Map ver. 7.0 (Definiens, Munich, Germany), and stored as RPG images in the jpg format with 648 × 432 pixels, resolution 150 × 150 dpi. Both conversion to the eight-bit images and their thresholding to the binary images, i.e., images having only 0 (black) and 255 (white) pixel values was performed using the Renyi Entropy filter of the open-source software Image J ver 1.48v (NIH, Bethesda, USA, http://imagej.nih.gov/ij/). The images were analyzed by the computer algorithms measuring the global fractal dimensions of the Renyi family, i.e. capacity dimension *D*_0_ and information dimension *D*_1_ (Benoit 1.3, True Soft, USA, http://www.trusoft-international.com/benoit.html), and correlation dimension *D*_2_ (Fractalyse 2.4, CNRS Universite de Franche-Comte, France, http://www.fractalyse.org). The values of the global fractal dimensions generated by the computer algorithms were verified against the geometrical model (Waliszewski, 2013; Waliszewski et al., 2014, 2015). Both the local fractal dimension and the local connected fractal dimension were measured in the same digitalized images using the open-source software Image J ver 1.48v and open-source plugin FracLac 2013 Janb420 by A. Karperien (http://imagej.nih.gov/ij/plugins).

### Statistical analysis

The statistical analysis including the ROC analysis was performed by Sigma Plot ver. 10.0 (Systat Software Inc., San Jose, USA, http://www.sigmaplot.com). The cluster analysis was performed by Mathematica ver. 10.0 (Wolfram Research Inc., USA) and Statistica ver. 8.0 (Statsoft, Oklahoma City, USA). The following aspects are important while analyzing a ROC curve. The ROC curve is a two dimensional graph. It represents a relationship between sensitivity of a statistical test (y−axis) in a function of 1–specificity of that test (x−axis). Sensitivity is defined as a rate of true positive events. The second quantity represents a rate of non-events that were falsely identified as positive events for the different cut-off values of a binary classifier, such as *D*_0_, *D*_1_, or *D*_2_, LFD, etc. Second, the closer the ROC curve follows the y-axis, and then the top border of the ROC diagram, the more accurate the statistical test. The area under the ROC curve (AUC) is a probability of the test accuracy, i.e., a probability that the classifier discriminates between the elements of two sets correctly. In particular, the AUC of 1.0 denotes a perfect relationship between the data that belong to a given category and those that do not. Third, the closer the ROC curve comes to the diagonal of the ROC diagram, the AUC is closer 0.5, what denotes that the test is not able to discriminate between two sets of data. It should be noted that a task of the re-stratification of prostate carcinomas on the basis of the quantitative characteristics of the spatial distribution of cancer cell nuclei by the fractal dimensions *D*_0_, *D*_1_, and *D*_2_ into the complexity classes is equivalent to the task of the identification the cut-off values of the best classifier, i.e., the classifier generating the AUC of 1.0 (Hill and Lewicki, 2006).

Cluster analysis is a data mining tool that uses a number of computer algorithms and statistical methods to sort elements of a set into groups (clusters) in such a manner that the elements belonging to the same cluster have a maximal degree of association and a minimal degree otherwise. In this work, a *v*-fold cross-validation algorithm was applied to determine the number of clusters in the data. Then, the *k*-means algorithm was applied to produce exactly *k* different clusters predicted by the first algorithm with the greatest possible distinction and maximal distances between those clusters. One examines here the means for each cluster on each dimension (parameter) to assess how distinct the *k*-means clusters are. Ideally, the means for all dimensions should be different, and the magnitude of the *F*-values from the analysis of variance performed on each dimension indicates how well the dimensions discriminate between clusters (Hill and Lewicki, 2006).

## Results

### The global capacity fractal dimension (*D*_0_) and the information fractal dimension (*D*_1_) discriminate well between the structural gleason classes (grades)

**Figure 1:**
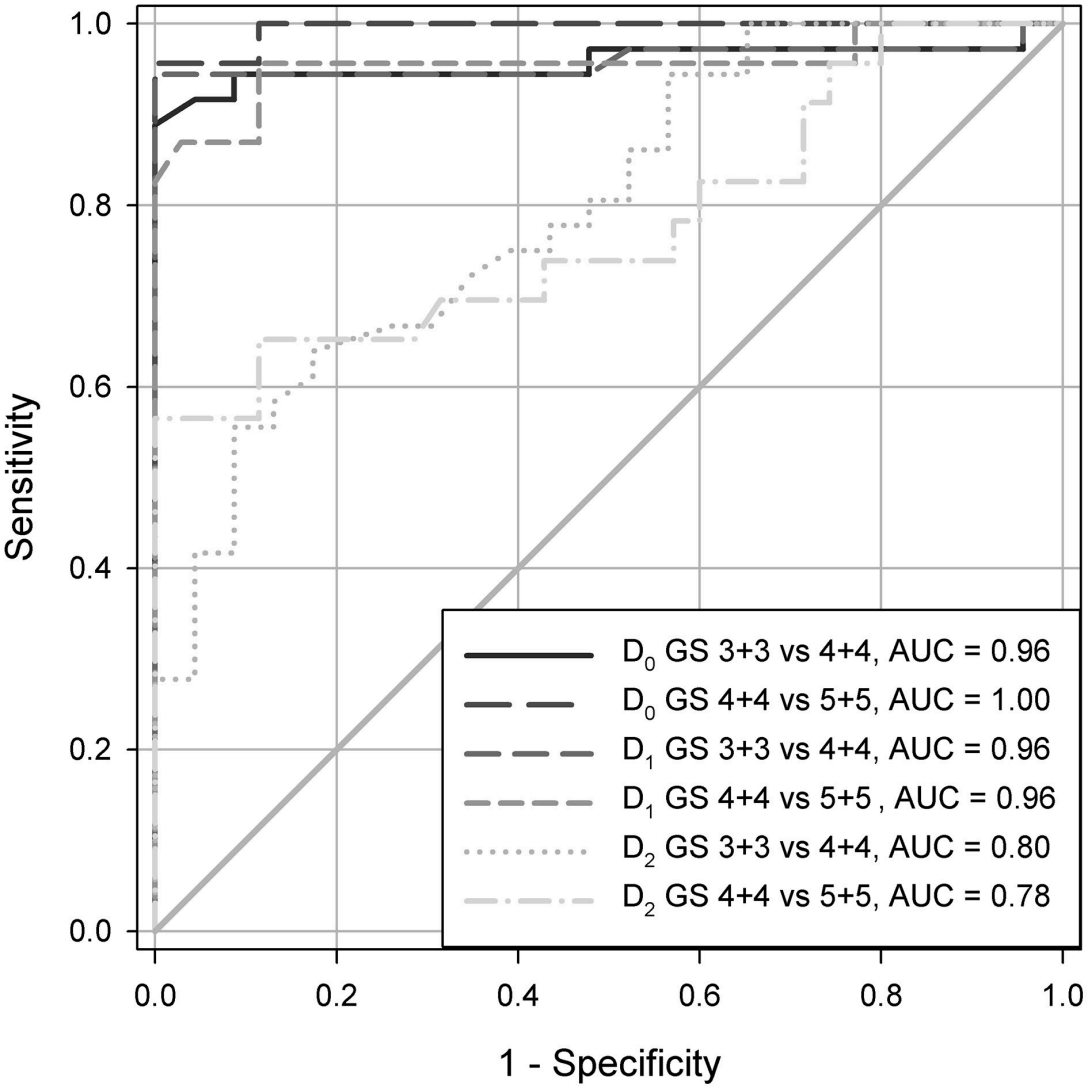
**The ROC analysis of prostate carcinomas with Gleason score 3+3, 4+4, and 5+5 of the test set demonstrating a discriminating power of the fractal dimensions *D*_0_, *D*_1_, and *D*_2_**. AUC stands for the area under the curve, and has values in a similar range as in the case of the reference set.

**Figure 2:**
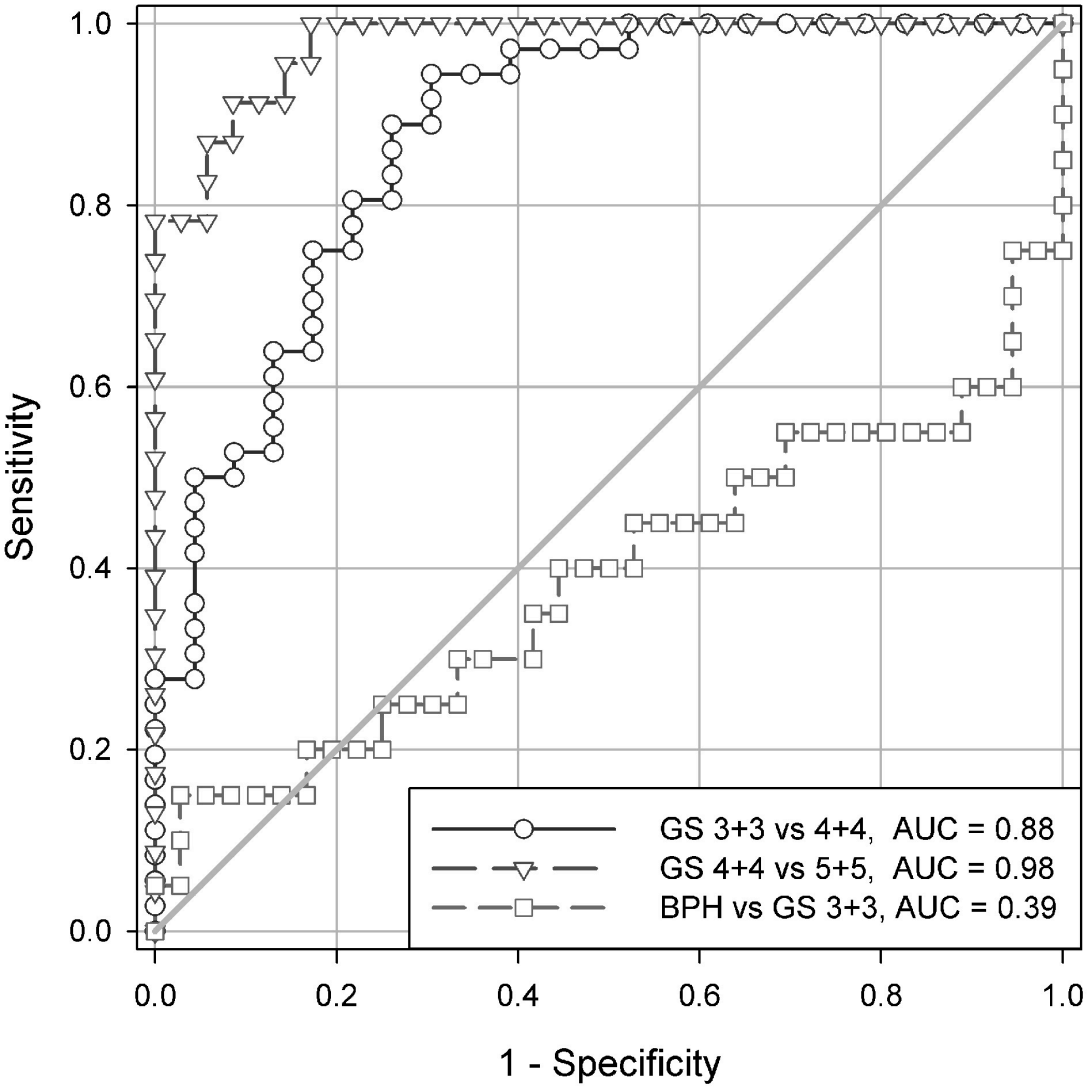
**The ROC analysis of carcinomas with Gleason score 3+3, 4+4, and 5+5 of the test set demonstrating a discriminating power of the local fractal dimensions LFD**. AUC stands for the area under the curve, and has values in a similar range as in the case of the reference set.

Both the global capacity and information fractal dimension, *D*_0_ and *D*_1_, have the sufficient statistical power to discriminate between the Gleason classes, that is the subsets of prostate carcinomas with the Gleason score 3+3, 4+4, or 5+5. Figures 1, 2 demonstrate that the dimension *D*_0_ was slightly better than the dimension *D*_1_ (Figure 1) and much better than the global correlation fractal dimension *D*_2_. In that latter case, the area under the curve (AUC) was almost 1.00 for the dimension *D*_0_ vs. 0.8 for the dimension *D*_2_. In addition, coefficient of correlation between the *D*_0_ and *D*_1_ was very high *R* = 0.963. The local fractal dimension, LFD, its variance, the local connected fractal dimension, LCFD, its variance, entropy *H*, and lacunarity λ were not able to discriminate between those classes at the statistically significant level (data not shown).

It is worth to notice that none of the complexity or the diversity measures can discriminate between the spatial distribution of cell nuclei in benign prostatic hyperplasia and in prostate carcinomas of the complexity class C1 containing carcinomas with the Gleason score 3+3 (compare Tables 1, **3**, and Figure 2). The mean values overlap (**Table 3**), and differences between those two groups of tissues are not statistically significant (*t*-Student test, *p* > 0.1).

**Table 1:** **Results of ROC analysis for the reference set (A) or the test set (B) of prostate carcinomas and their stratification into the structural Gleason classes**.

**Gleason classes**	**Sensitivity**	**Specificity**	**Cut off-value D_0_**	**AUC**	***p*-value**
**(A) REFERENCE SET**					
BPH vs. Gleason 3+3	0.50	0.40	1.5050	0.48	1.10
Gleason 3+3 vs. 4+4	1.00	1.00	1.6040	1.00	< 0.0001
Gleason 4+4 vs. 5+5	1.00	1.00	1.6910	1.00	< 0.0001
**(B) TEST SET**					
BPH vs. Gleason 3+3	0.50	0.51	1.5130	0.50	0.96
Gleason 3+3 vs. 3+4	0.63	0.67	1.5290	0.64	< 0.09
Gleason 3+4 vs. 4+3	0.78	0.67	1.5580	0.79	0.008
Gleason 4+3 vs. 4+4	0.75	0.78	1.6160	0.85	0.0008
Gleason 4+4 vs. 4+5	0.56	0.50	1.6400	0.47	1.27
Gleason 4+5 vs. 5+4	0.86	0.70	1.6870	0.84	< 0.0001
Gleason 5+4 vs. 5+5	0.80	0.66	1.7610	0.83	< 0.0001

BPH stands for benign prostatic hyperplasia. Data for benign prostatic hyperplasia cases were added to demonstrate that the approach excludes the automated diagnosis of prostate carcinoma. AUC denotes the area under the curve in the ROC analysis performed at a given p-value. Please, note that the set used in this study was larger and independent of the set used in the previous analysis (Waliszewski et al., 2015).

### The gleason classes and complexity classes are not identical

Table 1 contains results of ROC analysis for two sets of prostate carcinomas. The first one is a reference set. It contains carefully selected carcinomas with relatively homogeneous structure. Those carcinomas were stratified into three distinct structural classes with the Gleason score 3+3, 4+4, or 5+5. The spatial distribution of cancer cell nuclei in those carcinomas was also characterized quantitatively by the global fractal dimensions *D*_0_, *D*_1_, and *D*_2_. The ROC analysis enabled calculation of the cut-off *D*_0_-values that defined the numerical limits of those structural Gleason classes (see Table 1). As it turned out, even very careful histological evaluation of tumor structure brought together cases with a variety of infinitesimal structural alterations that could not be defined unambiguously. In consequence, each structural Gleason class of the reference set contained a low number of carcinomas with the quantitative characteristic typical of the other classes. Those cases were re-stratified using the cut-off *D*_0_-values for the structural Gleason classes as a frame of reference. After that operation, the new cut-off *D*_0_-values were calculated. They define the complexity classes C1, C4, and C7 (compare Table 1A with Table 2A).

**Table 2:** **Results of the ROC analysis for the re-stratified reference set (A) or the re-stratified test set (B) of prostate carcinomas**.

**Complexity classes**	**Sensitivity**	**Specificity**	**Cut off-value *D*_0_**	**AUC**	***p*-value**
**(A) REFERENCE SET**					
C1 vs. C4	1.00	1.00	1.5980	1.00	< 0.0001
C4 vs. C7	1.00	1.00	1.7000	1.00	< 0.0001
**(B) TEST SET**					
C1 vs. C2	1.00	1.00	1.5450	1.00	< 0.0001
C2 vs. C3	1.00	1.00	1.5820	1.00	< 0.0001
C3 vs. C4	1.00	1.00	1.6270	1.00	< 0.0001
C4 vs. C5	1.00	1.00	1.6490	1.00	< 0.0001
C5 vs. C6	1.00	1.00	1.6980	1.00	< 0.0001
C6 vs. C7	1.00	1.00	1.7640	1.00	< 0.0001

*The cut-off D_0_-values define the seven classes of complexity. Those classes of equivalence contain prostate carcinomas with the D_0_-values within the expected interval ranges as previously reported (Waliszewski et al., 2015). The complexity classes overlap to some extent with the structural Gleason classes. The average discrepancy in this study approximates 53%. AUC denotes the area under the curve in the ROC analysis performed at a given p-value. Please, note that the set used in this study was larger and independent of the set used in the previous analysis (Waliszewski et al., 2015)*.

### The cut-off *D*_0_-values of the structural gleason classes 3+4, 4+3, 4+5, or 5+4 are located between the values for the structural gleason classes 3+3, 4+4, or 5+5

The test set contains prostate carcinomas representing all Gleason scores. Those carcinomas were chosen randomly from the archive (Table 1B). The cut-off *D*_0_-values for the structural classes Gleason 3+3, 4+4, or 5+5 in that set are close to those of the reference set if analyzed without the structural classes 3+4, 4+3, 4+5, and 5+4. The same is true of the AUC values (Figures 1, 2) as well as sensitivity and specificity (data not shown). It appears that human eye deals much better with the evaluation of the distinct homogeneous structures rather than with the heterogeneous ones, where the number of details is greater, more difficult to grasp, and to set in order.

If the structural classes 3+4, 4+3, 4+5, and 5+4 are taken into consideration, the cut-off *D*_0_-values locate them between the values for the structural classes Gleason 3+3, 4+4, or 5+5. The *D*_0_ intervals defining those heterogeneous classes are smaller now (compare with Tables 1A,B). However, both sensitivity and specificity are not equal 1.00. The AUC values are also far from 1.0. This is because all structural classes of the test set including those with seemingly homogeneous carcinomas contain some number of cases with complexity measures typical of the other structural Gleason classes. As expected, those “impure” cases were re-stratified according to the *D*_0_-cut-off values, as described in the next paragraph, to the adjacent complexity classes exclusively.

### Re-stratification of prostate carcinomas into the seven classes of equivalence

Re-stratification puts aside a problem of accuracy of the subjective evaluation as well as troubles connected with a matching of tumor structure under examination to the grade definition. This was achieved by the application of the cut-off *D*_0_-values of the re-stratified reference set (see Table 2A). Carcinomas with the quantitative characteristics that did not match well the characteristics of the other carcinomas of the same class, nor of the adjacent class were identified and clustered in the novel classes of equivalence defined by the novel *D*_0_-values. As a result, carcinomas of the test set were re-stratified to the seven classes of complexity, i.e., C1, C2, C3, C4, C5, C6, and C7 (Table 2B). Table 3 provides a percentage of cases of each structural Gleason class that was a subject to re-stratification. This was from 30 to 70% of cases per structural class.

**Table 3:** **The values of the mean, median and standard deviations for the complexity or diversity measures in the re-stratified test set of prostate carcinomas**.

		**Staging Grading PSA ng/ml**	**pT1b - pT2a Gleason 6 PSA < 10**	**pT2b-pT2c Gleason 7-8 10 < PSA < 20**			**pT3a-pT3b Gleason 9-10 PSA > 20**		
		**Gleason Class**	**GS 3+3**	**GS 3+4**	**GS 4+3**	**GS 4+4**	**GS 4+5**	**GS 5+4**	**GS 5+5**
			***n* = 70**	***n* = 18**	***n* = 14**	***n* = 23**	***n* = 28**	***n* = 20**	***n* = 35**
**Complexity/Diversity Measures**		**BPH**	**Class C1**	**Class C2**	**Class C3**	**Class C4**	**Class C5**	**Class C6**	**Class C7**
		***n* = 20**	***n* = 60**	***n* = 20**	***n* = 37**	***n* = 16**	***n* = 23**	***n* = 29**	***n* = 23**
*D*_0_	Mean	1.5038	1.4836	1.5659	1.6035	1.6383	1.6684	1.7318	1.7986
	Median	1.5154	1.4895	1.5665	1.6023	1.6378	1.6660	1.7287	1.7944
	SD	0.0902	0.0490	0.0125	0.0125	0.0066	0.0138	0.0199	0.0241
*D*_1_	Mean	1.5222	1.5114	1.5673	1.6081	1.6474	1.6703	1.7282	1.7651
	Median	1.5321	1.5190	1.5700	1.6029	1.6403	1.6620	1.7286	1.7570
	SD	0.0936	0.0445	0.0250	0.0247	0.0232	0.0260	0.0221	0.0237
*D*_2_	Mean	1.5342	1.5525	1.6426	1.6657	1.7046	1.7069	1.7575	1.8107
	Median	1.5488	1.5640	1.6466	1.6687	1.6964	1.7144	1.7625	1.7993
	SD	0.0734	0.0977	0.0634	0.0586	0.0641	0.0511	0.0357	0.0452
LFD	Mean	1.6310	1.5109	1.5396	1.6529	1.6992	1.6897	1.7644	1.8225
	Median	1.6310	1.5564	1.5751	1.6492	1.6946	1.6998	1.7716	1.7989
	SD	0.0792	0.1447	0.2129	0.1497	0.1342	0.1236	0.0821	0.0552
CV	Mean	0.0939	0.0631	0.0582	0.0594	0.0761	0.0817	0.0780	0.0450
LFD	Median	0.0829	0.0677	0.0370	0.0466	0.0580	0.0629	0.0696	0.0450
	SD	0.0357	0.0253	0.0507	0.0453	0.0522	0.0502	0.0356	0.0144
LCFD	Mean	1.4507	1.4908	1.5716	1.6106	1.5663	1.6310	1.7051	1.7212
	Median	1.5223	1.5256	1.5625	1.6351	1.5815	1.6401	1.7369	1.7348
	SD	0.2063	0.1353	0.1665	0.2110	0.2639	0.1935	0.1313	0.1432
CV	Mean	0.1916	0.1403	0.1421	0.1074	0.1462	0.1002	0.1085	0.1154
LCFD	Median	0.1724	0.1261	0.1037	0.0973	0.1664	0.0944	0.0898	0.1178
	SD	0.0514	0.0473	0.0526	0.0671	0.0831	0.0687	0.0543	0.0609
*H*	Mean	0.5760	0.5638	0.6946	0.7594	0.7866	0.8457	0.9361	0.9500
	Median	0.5775	0.5803	0.7007	0.7504	0.7944	0.8451	0.9369	0.9861
	SD	0.1234	0.1072	0.0376	0.0337	0.0533	0.0219	0.0253	0.0661
λ	Mean	0.8516	0.8597	0.8125	0.7656	0.7527	0.7205	0.6284	0.6108
	Median	0.8515	0.8552	0.8141	0.7661	0.7507	0.7210	0.6095	0.5678
	SD	0.0524	0.0370	0.0215	0.0309	0.0367	0.0183	0.0405	0.0843

D_0_ denotes the global capacity fractal dimension, D_1_ stands for the global information fractal dimension, D_2_ is the global correlation fractal dimension, LFD is the local fractal dimension, LCFD is the local connected fractal dimension, CV-LFD stands for the LFD variation, CV-LCFD stands for the LCFD variance, H is the Shannon entropy, λ is lacunarity. The gray color denotes the existence of statistically significant differences between the complexity classes at p < 0.0001, t-Student test. The coefficient of correlation between the D_0_-values and D_1_-values is 0.9623.

**Figure 3:**
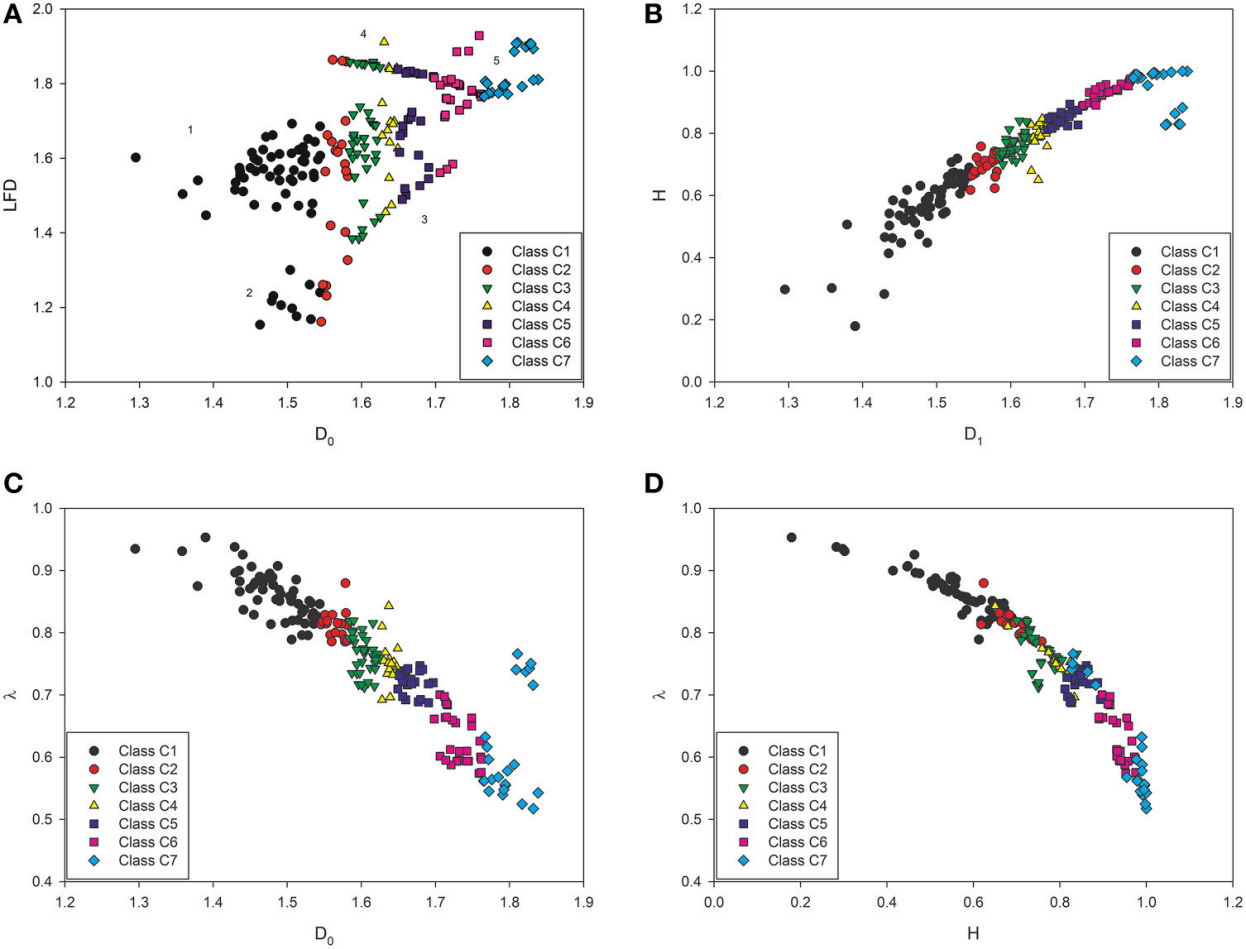
**Two dimensional scatter plots showing all prostate carcinomas of the test set stratified according to a number of relationships between the parameters: (A) (*D*_0_, LFD), (B) (*D*_1_, *H*), (C) (*D*_0_, λ), and (D) (*H*, λ)**. Differences between the complexity classes defined by the *D*_0_ or the *D*_1_ are statistically significant at *p* < 0.0001 (Table 3). **(A)** The LFD divides the complexity classes into the five subsets (clusters 1–5 are marked by the numbers; compare Figure 4A). Those clusters contain carcinomas with different values of the LFD, and, therefore, with different intercellular connectivity. In particular, 10 carcinomas of the class C1 (black circles) and four carcinomas of the class C2 (red circles) compose a cluster two with the lowest values of both the *D*_0_ and the LFD (Figure 4A), and the lowest aggressiveness. The values are close to those of benign prostatic hyperplasia. The cluster five represents high-grade carcinomas with the largest values of both the *D*_0_ and the LFD, the largest complexity, and the largest tumor aggressiveness (red squares and blue diamonds). **(B)** The dimension *D*_1_ is as strong classifier as the *D*_0_, and divides the set of prostate carcinomas in the same classes of equivalence. Although the values of the Shannon entropy *H* overlap between the classes, and, therefore, cannot play a role of the independent classifier, they characterize the spatial distribution of cancer cell nuclei in each class unequivocally if coupled with the *D*_0_ or *D*_1_-dimension. The coefficient of correlation for this relationship is 0.950. **(C)** The values of lacunarity λ overlap between the classes of equivalence; however, a combination of this diversity measure with the *D*_0_ or *D*_1_-dimension allows the unequivocal classification of the underlying spatial distribution of cancer cell nuclei. The coefficient of correlation for this relationship is −0.902. The statistical linearity of this relationship confirms in the independent way the existence of fractal structure in the spatial distribution of cancer cell cell nuclei. **(D)** Neither the Shannon entropy *H* nor lacunarity λ are strong classifiers. The values overlap between the classes of equivalence. A small subset of carcinomas of the class C7 overlaps with carcinomas of the class C5. This results from a lower number of cells in cellular infiltrates. However, this relationship is tri-linear. The coefficients of correlation for this relationship are −0.911, −0.650, and −0.922 for *H* < 0.7, 0.7 < *H* < 0.9, and *H* > 0.9, respectively. The existence of tri-linearity suggests that some kind of qualitative transitions occur in natural history of prostate cancer between the class C2 and C3 as well as the class C5 and C6.

It should be emphasized that all complexity classes are unequivocally defined by the cut-off *D*_0_-values. Therefore, the complexity classes contain only those prostate carcinomas that have the spatial distribution of cancer cell nuclei characterized by the similar values of the dimension *D*_0_ and hold a condition that the AUC in the ROC analysis equals 1.0. As expected, an identical re-stratification can be obtained with *D*_1_-values (compare Figure 3B). Each complexity class contains elements of the corresponding structural Gleason class and some carcinomas of the other structural Gleason classes with the *D*_0_-values within the range for that class. For example, Figure 3A presents each carcinoma of the test set as a point on the 2D-scatter plot with the *D*_0_ and the LFD as variables. Each carcinoma belongs to one out of seven classes of equivalence according to the *D*_0_-values. Table 3 shows the statistically significant differences between all complexity classes for the *D*_0_, and the *D*_1_ (*p* < 0.0001, *t*-Student test). This finding confirms a choice of the cut-off *D*_0_ or *D*_1_-values as a criterion for the re-stratification.

### A relationship between the global capacity dimension *D*_0_ and lacunarity λ or the global information dimension *D*_1_ and entropy *H* or is linear

The relationship between the *D*_0_ and λ is given by linear equation (8):

(8) $$\lambda =2.0701-0.8145D_{0}$$

The coefficient of correlation R for that relationship was -0.902. As it can be seen in Figure 3C, a given value of lacunarity λ may be associated with different values of the *D*_0_ dimension and different corresponding spatial distributions of cancer cell nuclei that belong to the neighbor classes of equivalence. Hence, the λ value is not a good classifier. However, if this parameter is coupled with the *D*_0_-value, it characterizes the spatial distribution of cancer cell nuclei unequivocally.

A similar capability possesses a pair (*D*_1_, *H*). As expected, there was a linear relationship between the global information fractal dimension *D*_1_ and entropy *H* given by the following statistical equation (9)

(9) $$H=-1.4280+1.3511D_{1}$$

Figure 3B demonstrates a distribution of carcinomas around the linear curve defined by Equation (9). The coefficient of correlation *R* for that relationship was 0.950. The linear relationship between the global information dimension as a complexity measure of cell nuclei clustering and the Shannon entropy as a diversity measure of information content validates in the independent manner the choice of the classes of equivalence. The *D*_1_ is more accurate classifier than the Shannon entropy *H* (Figure 3B). Some carcinomas of the class C3, C4, C5, C6, or C7 have entropy values that overlap with the values of the cases belonging to the adjacent classes. In particular, carcinomas of the class C7 are composed of cellular infiltrates only. Some of them have, however, a much lower number of infiltrating cells. Entropy of those carcinomas is lower, respectively (see Figure 3B, Class C7, light blue diamond). The values of lacunarity for those cases overlap with those for carcinomas of the class C5 (see Table 3 and Figure 3D).

A statistical relationship between lacunarity λ and the Shannon entropy *H* is more complex. This relationship is described by three linear equations that are defined for different intervalls of the *H*-values. The first one is given by equation (10)

(10) λ=1.0301−0.3057H

and holds for *H* = < 0.7. The coefficient of correlation *R* = −0.911.

The second one is given by Equation (11)

(11) λ=1.1625−0.5224H

and holds for 0.7 < *H* < 0.9. The coefficient of correlation *R* = −0.650.

The third one is given by Equation (12)

**Figure 4:**
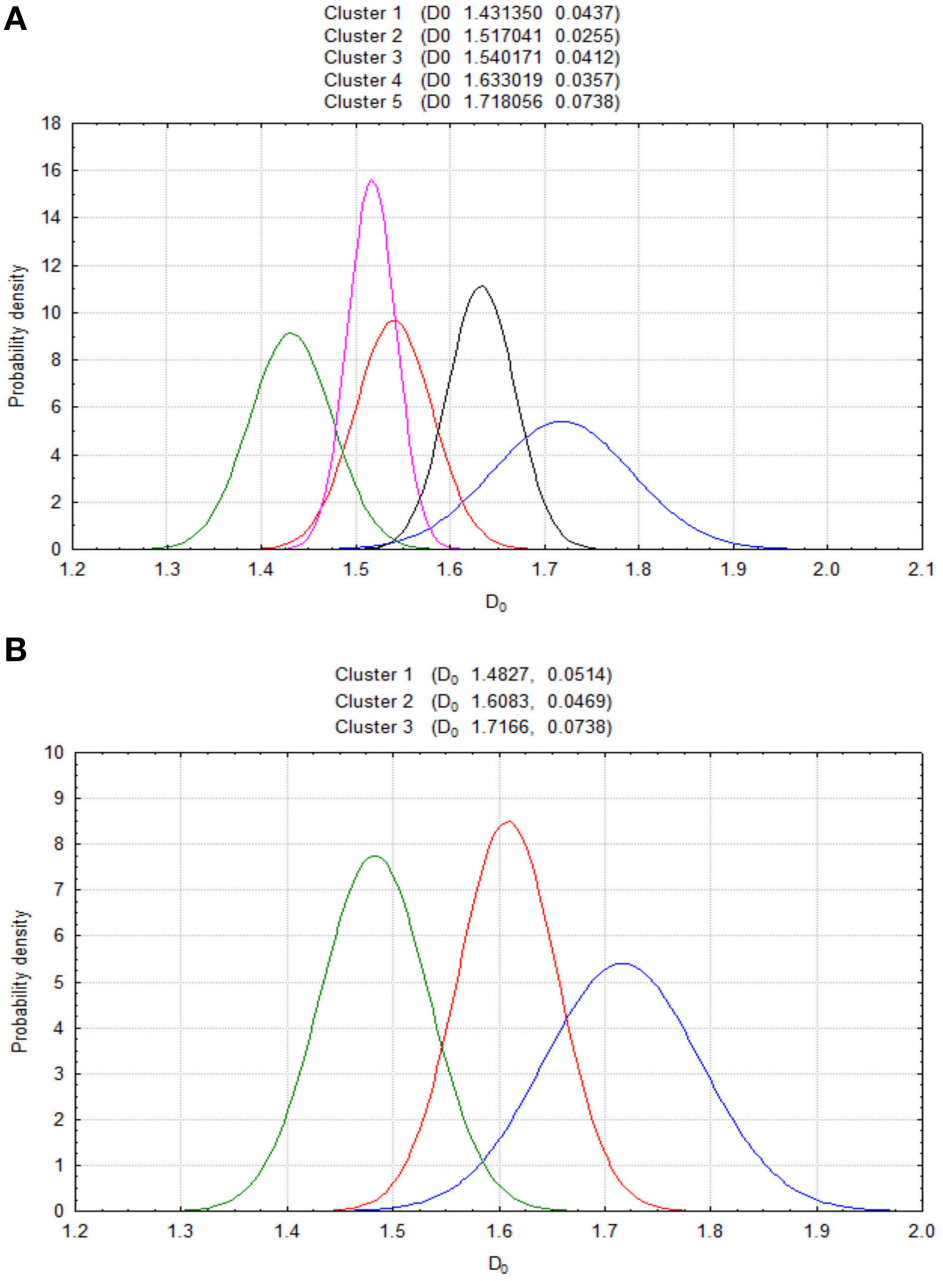
**Results of cluster analysis for the test set of prostate carcinomas with the dimensions *D*_0_ and LFD (A) (ANOVA *p* < 0.001, *F*_*D*0_ 45799, F_LFD_ 31935) and the parameters *D*_0_, LFD, LCFD, *H*, λ (B) (ANOVA *p* < 0.001, *F*_*D*0_ 75800, *F*_LFD_ 25289, *F*_LCFD_ 13202, *F*_*H*_ 7925, *F*_λ_ 16102)**. The increment of the number of parameters from two to five reduces a number of clusters from seven to three. The objective analysis can identify in that way carcinomas with low-, intermediate-, and high complexity of the spatial distribution of cancer cell nuclei corresponding to low-, intermediate- or high tumor aggressiveness. The following conditions can be defined: for *D*_0_ < 1.5820, LFD < 1.3, LCFD > 1.5, *H* < 0.7 and λ > 0.8, the class C1 or C2 contains low-complexity, low-grade carcinomas exclusively; for *D*_0_ > 1.6980, LFD > 1.7644, LCFD > 1.7051, *H* > 0.9, and λ < 0.7, the class C6 or C7 contains high-complexity, high-grade carcinomas only.

(12) λ=1.7503−1.199H

and holds for *H* > 0.9. The coefficient of correlation *R* = −0.901. Those relationships characterize well only the spatial distribution of cancer cell nuclei in prostate carcinomas with very low or very high diversity as measured by the Shannon entropy. Prostate carcinomas with intermediate diversity cannot be stratified with sufficient accuracy (compare Figures 3D,4B). The values of the pairs (*H*, λ) overlap for many cases that belong to different classes of equivalence defined by the *D*_0_ or the *D*_1_ (Figure 3D). Figure 3A shows that the spatial distributions of cancer cell nuclei in many prostate carcinomas are unique as represented by different values of the pair (*D*_0_, LFD) (Figure 3A). Those values are not identical owing to multifractality. Simultaneously, many of those carcinomas have the same or almost identical values of the pair (*D*_1_, *H*) (Figure 3B) or (*H*, λ) (Figure 3D). Two different fractal dimensions denote the existence of two different spatial distributions of cell nuclei. However, if the difference is not large, the Shannon entropy *H* or lacunarity λ may be identical owing to variability in cell numbers or their clustering.

### The co-application of the complexity and diversity measures in the evaluation of the spatial distribution of cancer cell nuclei in prostate carcinomas

The cluster analysis by the *k*-means algorithm shows that the co-application of both complexity and diversity measures, *D*_0_ (*D*_1_), LFD, LCFD, *H*, and λ leads to a reduction of a number of clusters from seven to three (Figure 4B). Since low grade prostate carcinomas possess well-preserved glandular structure, the values of all the above measures but λ are also low. The reduction of the number of clusters identifies some carcinomas of the complexity classes C1 and C2 as elements of the first cluster with the lowest values of those parameters, and, therefore, the lowest tumor aggressiveness. Similarly, one can define the quantitative criteria for carcinomas with the highest values of those parameters but λ, and the highest aggressiveness.

The mean LFD values do not increase along the complexity classes monotonically (see Figure 3A and Table 3). Rather, those values increase along each stratum, that is, a subset composed of the classes C1 and C2 or C3 and C4, or C5 and C6, or C7 (Table 3). The mean LFD values can be identical for carcinomas of different complexity classes.

The cluster analysis indicates five such subsets of prostate carcinomas characterized by the pair (*D*_0_, LFD), in which the *D*_0_ is a measure of the global spatial distribution of cancer cell nuclei and the local fractal dimension LFD is a measure of intercellular connectivity (see Figures 3A,4A).

In the first subset, characterized by the parameters *D*_0_ < 1.5820 and LFD < 1.35, fourteen prostate carcinomas of the class C1 or C2 (low grade prostate carcinomas with mean PSA < 6.1 ng/ml, standard deviation 1.2 ng/ml, pT1b, pT1c, or pT2a, Gleason score 6, relatively homogeneous well-preserved glandular structure with glands distributed in tissue sparsely, without a clustering or crowding, none of those patients developed recurrence or metastases, or died owing to prostate cancer during the 5 years of follow-up) revealed very high intercellular connectivity. Those features are reflected by the value of the local connected fractal dimensions LCFD close to 1.5 (the scatter plot not shown), low values of entropy *H* < 0.7 and high values of lacunarity λ > 0.8 (compare Figures 3A–D). The low values of the fractal dimensions indicate that both global and local complexity in those carcinomas is low. In addition, the values of those fractal dimensions are quite close to the values obtained for benign prostatic hyperplasia (see Table 3). All those local and global features of the spatial distribution of cancer cell nuclei are associated with a low potential for growth and aggressiveness.

The second subset is composed of the low risk adenocarcinomas (mean PSA 8.3 ng/ml, standard deviation 2.6 ng/ml, Gleason score 6, pT1b, pT1c, or pT2a) stratified also to the classes C1 or C2. However, the second subset has the same values of the *D*_0_, *D*_1_, almost the same values of entropy *H* < 0.78 as well as lacunarity λ > 0.78, and 1.300 < LFD < 1.750. The values of the first four parameters denote that number of cells and their spatial configurations between those two sets are comparable. However, the values of the LFD indicate the existence of more advanced alterations in intercellular connectivity among adenocarcinomas of the second subset in spite of the preserved glandular structure, and, therefore, denote a higher risk of progression. Those LFD values denote very slow, yet active growth owing to the lower intercellular connectivity, and, therefore, increased probability of metastasizing in the course of time in comparison with the first subset. Those cancer patients belong to a “gray” zone, in which a follow-up without any additional therapy may work fine for some cases of the lower classes of complexity, such as C1, C2, or even for some intermediate-risk cases of the class C3 and C4 having the low values of the LFD.

The third and the fourth subset comprise intermediate risk prostate cancers of the complexity class C3, C4, C5 (mean PSA 12.7 ng/ml, standard deviation 2.2 ng/ml, Gleason score 7 and 8, pT2b-2c). In the third subset, there are carcinomas with 1.5820 < *D*_0_ < 1.6980 and the LFD < 1.8. Prostate carcinomas of the fourth subset have the same values of the *D*_0_ as in the third set, but the LFD > 1.8 (see Figure 3A) at 0.65 < *H* < 0.85, and 0.7 < λ < 0.82 (Figures 3B,C). Surprisingly, there are two adenocarcinomas of the complexity class C2 in that subset. Their mean LFD value approximate the value of 1.9, that is typical of prostate carcinomas without glandular structure and with the increased potential to metastasize. In spite of the stratification to the low class of complexity owing to the *D*_0_-value, such carcinomas with very low intercellular connectivity may have a high metastatic potential.

The fifth subset comprises high risk prostate cancers (mean PSA 24.3 ng/ml, standard deviation 3.1 ng/ml, Gleason score 9–10, stage T3a, the largest ratio of prostate carcinoma related death or metastasis formation within 5 years from the surgical treatment 0.442) of the complexity class C6 or C7 with *D*_0_ > 1.6980 and the LFD > 1.8, *H* > 0.9, and λ < 0.7. The large values of the *D*_0_ and *H* indicate a significantly increased cell number owing to the intense cell proliferation and rapid growth. Since images are covered almost completely by cancer cells, and become almost symmetrical, the λ-values are low. The high values of the LFD indicate the existence of a very low intercellular connectivity. Cells fill up the entire available tissue space without any clustering, and, therefore, have the largest potential for metastasis formation.

## Discussion

The classes of equivalence defined by the cut-off values of the global capacity dimension *D*_0_ reported previously (Waliszewski et al., 2015) have been validated. The other measures but the *D*_1_ failed to define the same classes of equivalence. However, the co-application of all the measures allowed the formulation of two quantitative criteria identifying low or high aggressive prostate carcinomas.

A novel, quantitative strategy for the evaluation of tumor aggressiveness excludes the automated diagnosis of prostate carcinoma. The complexity or diversity measures were not able to discriminate between the spatial distribution of cell nuclei in benign prostatic hyperplasia, a common lesion in human prostate, and carcinomas with a well-preserved glandular structure (see Table 3). This approach implies a necessity to examine the entire prostate carefully, what does not seem to be the case in the routine practice (True, 1994). Indeed, the largest value of the fractal dimension *D*_0_ or *D*_1_ determines the stratification of carcinomas into a given class of equivalence.

Results of this study suggest that the number of the subjective Gleason grades of tumor aggressiveness could be decreased from seven to three without a loss of clinically important information, that is, to the low, intermediate, or high grade. The identical reduction could be done for a number of the objective classes of equivalence. Table 1 indicates that pathologists can distinguish quite well between the relatively homogeneous patterns with the Gleason score 3+3, 4+4, or 5+5 (see the reference set). However, heterogeneous patterns defined as the Gleason score 3+4, 4+3, 4+5, or 5+4 were classified with much greater inaccuracy (see the test set). The classes of equivalence defined by the cut-off *D*_0_-values enable the quantitative evaluation of tumor aggressiveness of borderline cases with mixed tissue architecture; a frequent problem in pathology of prostate carcinoma (Dong et al., 2012). It should be noticed that the discrepancy between the subjective Gleason classification and the classification according to complexity of the spatial distribution of cancer cell nuclei was on average about 53% in this study; a value that is in the range of the intra- and interobserver variability (McLean et al., 1997; Allsbrook et al., 2001; Nguyen et al., 2004; van der Kwast et al., 2010; McKenney et al., 2011; Netto et al., 2011; Dong et al., 2012; Egevad et al., 2013; Scott Lucia et al., 2013; Berney et al., 2014).

The classes of equivalence, i.e., the complexity classes defined by the cut-off values of the *D*_0_ dimension (or the *D*_1_ dimension) were validated by analyzing the test set of carcinomas with sensitivity, specificity, and AUC equal 1.0 (Waliszewski et al., 2015). This set was larger and independent from the set analyzed in the previously published study (Waliszewski et al., 2015). The values of the global capacity fractal dimension *D*_0_ are identical with those previously reported (Waliszewski et al., 2015) (see Tables 2, 3, Figure 3). Those two dimensions appear to be the best classifiers of prostate carcinomas according to the spatial distribution of cancer cell nuclei. There is a very good correlation between the values of the *D*_0_ and the *D*_1_ (correlation coefficient *R* = 0.962). Those results were to be expected since both sets comprised different numbers of carcinomas with the similar patterns of tumor growth. As expected, the *D*_1_-values correlate very well with the values of the Shannon entropy *H*; a diversity measure of both randomness in the spatial distribution of cell nuclei and information content in images of a given class (*R* = 0.950; see Figure 3B). There also is some correlation between the values *H* and λ that changes depending on the *H* intervals (compare Equations 10–12). The λ values overlap between classes (see Figure 3D), and, therefore, cannot be applied for stratification as the independent classifier. The overlapping results from the non-bijective relationship between some configurations of cell nuclei and the measures applied (Waliszewski et al., 2015). In particular, two borderline spatial distributions of cancer cell nuclei may have identical values of the complexity measures, but different values of the diversity measures. In spite of that weakness, the diversity measures help to identify carcinomas with low or high diversity corresponding to carcinomas with the low or high grade (see Figures 3B,C). Each of those measures characterizes quantitatively different aspects of the spatial distribution of cancer cell nuclei in images of prostate carcinomas. While the *D*_0_ or the *D*_1_ enables the stratification of carcinomas into the seven classes of equivalence, that remain unchanged if the LFD, the LCFD, the Shannon entropy *H* or λ are added to the class characteristics (see Figure 3), the co-application of all the above-studied measures allows the stratification of the same carcinomas into just three distinct clusters with low tumor aggressiveness (class C1, C2, and some carcinomas of the class C3), intermediate one (some carcinomas of the class C3, C4, and C5) or high one (class C6 and C7) (compare Figure 4). The appropriate quantitative criteria are defined in the Sections Results: A Relationship between the Global Capacity Dimension *D*_0_ and Lacunarity λ or the Global Information Dimension *D*_1_ and Entropy *H* or is Linear; The Co-Application of the Complexity and Diversity Measures in the Evaluation of the Spatial Distribution of Cancer Cell Nuclei in Prostate Carcinomas.

The presented data indicate some change in intercellular connectivity at the interface between classes C2 and C3 as well as C5 and C6 as measured by the LFD; a complexity measure of cellular connectivity, that is, a measure of strength of intercellular interactions (Table 3, Figure 3A). This change can also be seen in the scatter plot (*H*, λ) (Figure 3D). The relationship between the Shannon entropy H and lacunarity λ; a diversity measure of cell clustering also changes at the interface between the above-mentioned classes and depends on the *H*-values (*R* = −0.911 for *H* < 0.7, *R* = −0.650 for 0.7 < *H* < 0.9, and − 0.901 for *H* > 0.9). Those changes correspond with the enhanced dynamics of tumor growth at the interface between low and intermediate risk cancers as well as intermediate and high risk cancers. It is possible that all those changes result from some genetic instability that gains influence on growth dynamics at some specific check-points in natural history of prostate cancer. Second, results of the analysis point out that prostate carcinomas with the spatial distributions of cancer cell nuclei holding the conditions *D*_0_ < 1.5820, LFD < 1.3, and LCFD > 1.5 are true low grade carcinomas. Those carcinomas possess a well-preserved, relatively homogeneous glandular structure that resembles the structure of the normal prostatic glands. There is some intermediate cell clustering at the low values of the LFD and, therefore, high intercellular connectivity in cell populations of those adenocarcinomas (Figure 3A). Hence, there is no high growth potential in those cellular populations. If *D*_0_ > 1.6980, LFD > 1.7644, and LCFD > 1.7051, then tumor aggressiveness is large. Cancer cells infiltrate the entire available tissue space, have low intercellular connectivity, do not interact each other, and compose passively multiple cell clusters; the overcrowding effect. This constellation of dimensions indicates the existence of a large growth potential in a cellular population. The cases with intermediate conditions compose a gray zone. Some of those carcinomas may do well under follow-up, especially those with the lower values of the LFD and LCFD. The other cases will certainly require some kind of therapy in the course of time. It is interesting, however, that there is no carcinoma with the low values of both local fractal dimensions. If the LFD increases, then intercellular connectivity decreases, and cells lose their capability to interact with each other. Therefore, cells in some carcinomas including those of the class C1, C2, C3, and C4 do not create clusters (the lower values of the LCFD), while many others still do (the larger values of the LCFD). The loss of capability for cell clustering at low values of the LCFD may also denote both the increased growth potential and risk of metastasis formation.

The increasing values of the *D*_0_ dimension denote that cellular proliferation in a given carcinoma is very extensive, and more and more cancer cells fill up the available tissue space. If cancer cell gather in some kind of glands or gland-like objects, the *D*_0_-values are lower than if there dominate cellular infiltrates in the image (compare the values of the *D*_0_ for the classes C1, C2 or C3 with those for the classes C6 or C7 in Table 3). The larger is the value of the *D*_0_ the higher is the complexity class and the corresponding subjective grade. The higher is the complexity class the higher is a number of cancer cells present in the image of prostate carcinoma. Cancer cells lose in the course of natural tumor evolution their cluster organization in a form of glands (high values of the *D*_1_, the LFD, and the LCFD and low values of λ), become more randomly distributed (high values of *H*), and reveal lower connectivity (high values of the LFD and the LFCD) (compare Figures 3A–D). The risk of local progression or metastasis formation is increased in those spatial distributions of cancer cell nuclei.

The existence of both the global and local fractal dimensions for a given spatial distribution of cancer cell nuclei implies that that spatial distribution underlies a scale-dependent power law. The existence of the power law means that the spatial distribution of cancer cell nuclei undergoes somehow ordered rather than utterly random changes in the course of natural history of the prostatic tumorigenesis. That order of events appears to comprise the gradual loss of intercellular connectivity in carcinomas of the higher complexity classes (see Table 3 and Figure 3). This may occur owing to the loss of expression of adhesion molecules or some other molecules with biologically important functions leading to alterations of intercellular interactions (Busch et al., 2002; Huang et al., 2009; Mathieu et al., 2013; Schrecengost and Knudsen, 2013). For the majority of carcinomas, the values of both the global and local fractal dimension are not identical (see Table 3 and Figure 3). That difference between the values of the global and local fractal dimensions indicates the existence of multifractals in the spatial distributions of cancer cell nuclei, that is, fractals that scale with multiple scaling rules and hold the condition *D*_0_ > *D*_1_ > *D*_2_ (reviewed in Lopes and Betrouni, 2009). Multifractality suggests that the final spatial distribution of cancer cells depends on both a variety of molecular events and long-range phenomena such as diffusion. The interactions may occur at different levels of the hierarchical tissue dynamic system (Waliszewski, 1997; Waliszewski et al., 1998, 2001). Those complex phenomena compose a new world to be explored.

Two diversity measures CV-LFD and CV-LCDF do not play a role in the stratification process (see Table 3). Those diversity measures apply to differences in measurements of the local fractal dimensions within each complexity class (Page, 2011). There is no monotonic increment of the values of those parameters that might enable the stratification process. However, entropy, the other diversity measure, appears to be useful in the additional characterization of the spatial distribution of cell nuclei (Equations 2, 4, 5 and Figure 3B). Even though the local fractal dimensions in each complexity class that relate to single cancer cell nuclei are not diverse, cancer cells as a population form diverse dynamic spatial structures of the higher order, such as cell clusters (see Figure 3A). Those cell clusters can be characterized locally by the values of the local connected fractal dimension, entropy, and lacunarity. In addition, the LFD identifies cells with the lowest intercellular connectivity, and, therefore, having the maximal metastatic potential (see Figure 3A; Waliszewski, 1997, 2005, 2009, 2013; Waliszewski et al., 1998, 2001; Waliszewski and Konarski, 2001). Computer simulations of a model considering the evolution of cooperation in a spatial setting obtained by Nowak and May show clearly the existence of a relationship between emergent diversity and complexity (Nowak and May, 1992, 1993). The existence of that relationship in prostate carcinomas can be seen in equation 5 and Figure 3B.

To summarize, the following findings must be reiterated. First, the classes of equivalence for prostate carcinomas by the cut-off *D*_0_-values were validated. Second, no other measure studied but the *D*_1_ can define the same classes of equivalence. Third, carcinomas with low complexity of the spatial distribution of cancer cell nuclei reveal low biological aggressiveness, and vice versa. Some pairs of the parameters (*D*_0_, LFD), (*D*_0_, *H*) or (*D*_0_, λ) or (*D*_1_, LFD), (*D*_1_, *H*) or (*D*_1_, λ) characterize well complexity of the spatial distribution of cancer cell nuclei in each class of equivalence. The tri-linear course of the relationship (*H*, λ) suggests that among carcinomas of the classes C2 and C3 as well as C5 and C6 occurs some kind of a qualitative transition that implicates changes in tumor aggressiveness. Fourth, the co-application of all complexity and diversity measures reduces a number of clusters, but still enables the identification of carcinomas with low or high complexity of the spatial distribution of cancer cell nuclei. Those findings suggest the number of both the subjective Gleason grades and the objective classes of equivalence could be decreased from seven to three.

## Author contributions

PW: all ideas, construction of the prostate data bank, digitalization of images, statistical analyses, writing of the manuscript.

### Conflict of interest statement

The author declares that the research was conducted in the absence of any commercial or financial relationships that could be construed as a potential conflict of interest.
